# Primary ovarian carcinoid arising in associated mature cystic teratoma

**DOI:** 10.1186/s12905-022-01660-x

**Published:** 2022-03-17

**Authors:** Wanyu Zhang, Qiongrong Chen

**Affiliations:** Department of Pathology, Zhongnan Hospital of Wuhan University, No. 169 Donghu Road, Wuhan, 430071 Hubei Province People’s Republic of China

**Keywords:** Primary ovarian carcinoid, Neuroendocrine tumor, Mature cystic teratoma

## Abstract

**Background:**

Primary ovarian carcinoid is a very rare ovarian low-grade neuroendocrine tumor, accounting for about 0.1% of all ovarian neoplasms.

**Case presentation:**

We reported a case of primary ovarian carcinoid arising from a mature cystic teratoma in a 50-year-old woman. Intraoperative frozen section of left ovarian mass was assessed and a malignant epithelial tumor was considered. Morphologically, the main tumor was composed of cells forming trabeculae, and mature cystic teratoma was observed adjacent to the main part. Immunohistochemistry revealed that the trabecular cells were diffuse positive for pan Cytokeratin, CD56 and synaptophysin with low Ki-67 index (about 1%).

**Conclusions:**

Careful morphological observation combined with appropriate accessory examination are essential for the diagnosis of primary ovarian carcinoid arising from mature cystic teratoma. In addition, the classification criteria of the primary ovarian neuroendocrine tumor are discussed.

## Background

Carcinoid is a kind of low-grade neuroendocrine tumor (NET) named NET G1 in the 5^th^ edition of the World Health Organization (WHO) classification of tumors, in volume 1 and volume 5, respectively [1, 2]. It usually occurs in the gastrointestinal and respiratory tracts. Ovarian carcinoid (OC) [3], also named as strumal carcinoid in the 4^th^ WHO classification of tumors of female reproductive organs [4], is a very rare primary ovarian tumor, accounting for approximately 0.1% of all ovarian neoplasms [5]. Only 15% of these reportedly exist in pure form, with the remainder featuring teratomatous components such as struma ovarii or dermoid cysts [6]. Here, we report a case of primary ovarian carcinoid arising in association with a mature cystic teratoma.

## Case presentation

A 50-year-old perimenopausal woman presented with intermittent abdominal pain, vaginal contact bleeding, mild headache and dizziness. Transvaginal ultrasound revealed a mass with a diameter of 2.5 cm in her left adnexal region. Gynecological magnetic resonance imaging (MRI) showed an abnormal signal nodule in the left adnexal area, which was considered a neoplastic lesion. The tumor marker showed a raised neuron-specific enolase (NSE). No other abnormalities were found by imaging examination and the patient had no genetic history. Intraoperative frozen section of the left ovarian mass was assessed and a malignant epithelial tumor was considered. The patient then underwent a total abdominal hysterectomy, bilateral salpingo-oophorectomy, pelvic lymphadenectomy and omentectomy. The patient recovered well and discharged 3 days after surgery, and was well on routine follow-up.

**Fig. 1:**
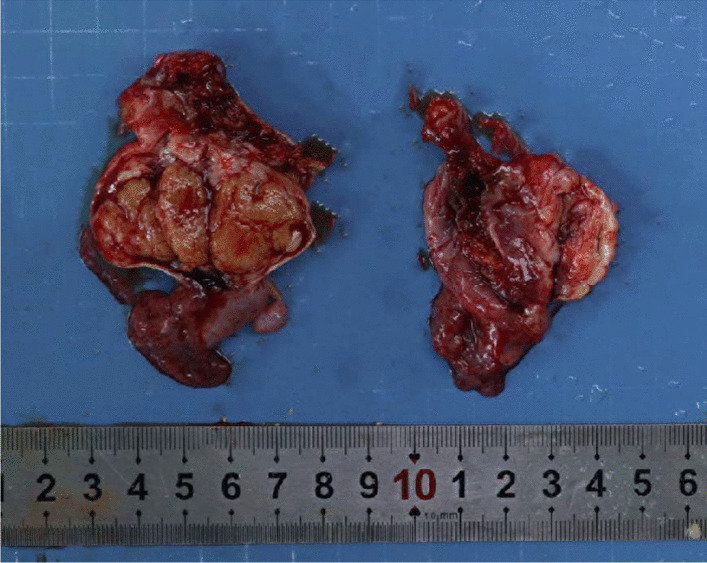
There was a mass measuring 2.5 cm × 1.6 cm × 1.2 cm in the left ovary, and the cut surface looked yellow and solid

**Fig. 2:**
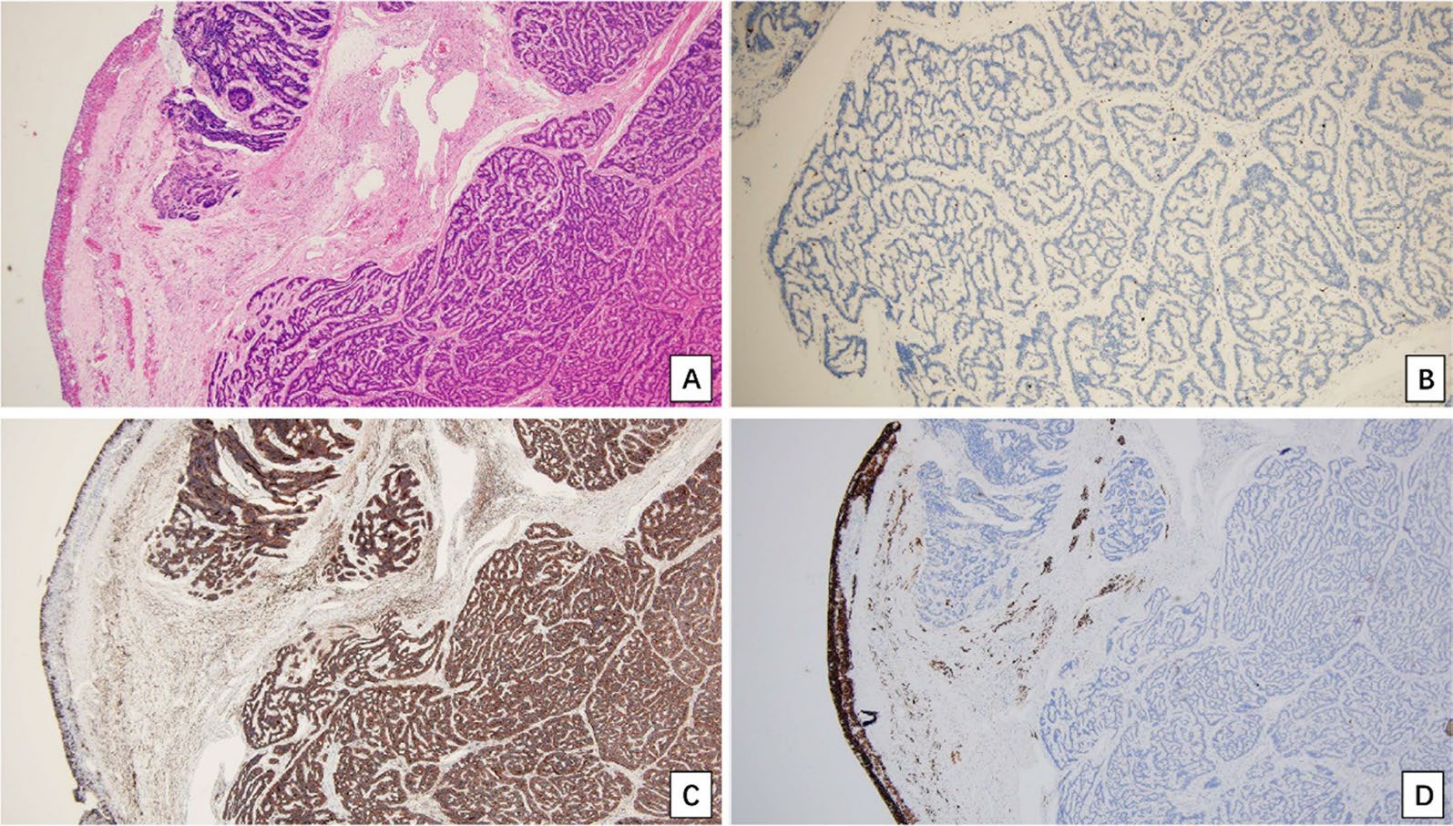
The representative morphological and immunohistochemical features of the primary ovarian carcinoid. **A** H&E staining showed the tumor cells forming parallel ribbons, cords, or trabeculae. **B** The Ki-67 labeling index of the tumor cells was very low (about 1%); **C** the tumor cells were strongly positive for SYN; **D** the tumor cells were negative for α-inhibin, but the internal control was positive (left, cyst wall of ovary follicular cyst). (**A** H&E, × 40; **B**, **C**, **D** IHC with hematoxylin counterstain, × 40)

**Fig. 3:**
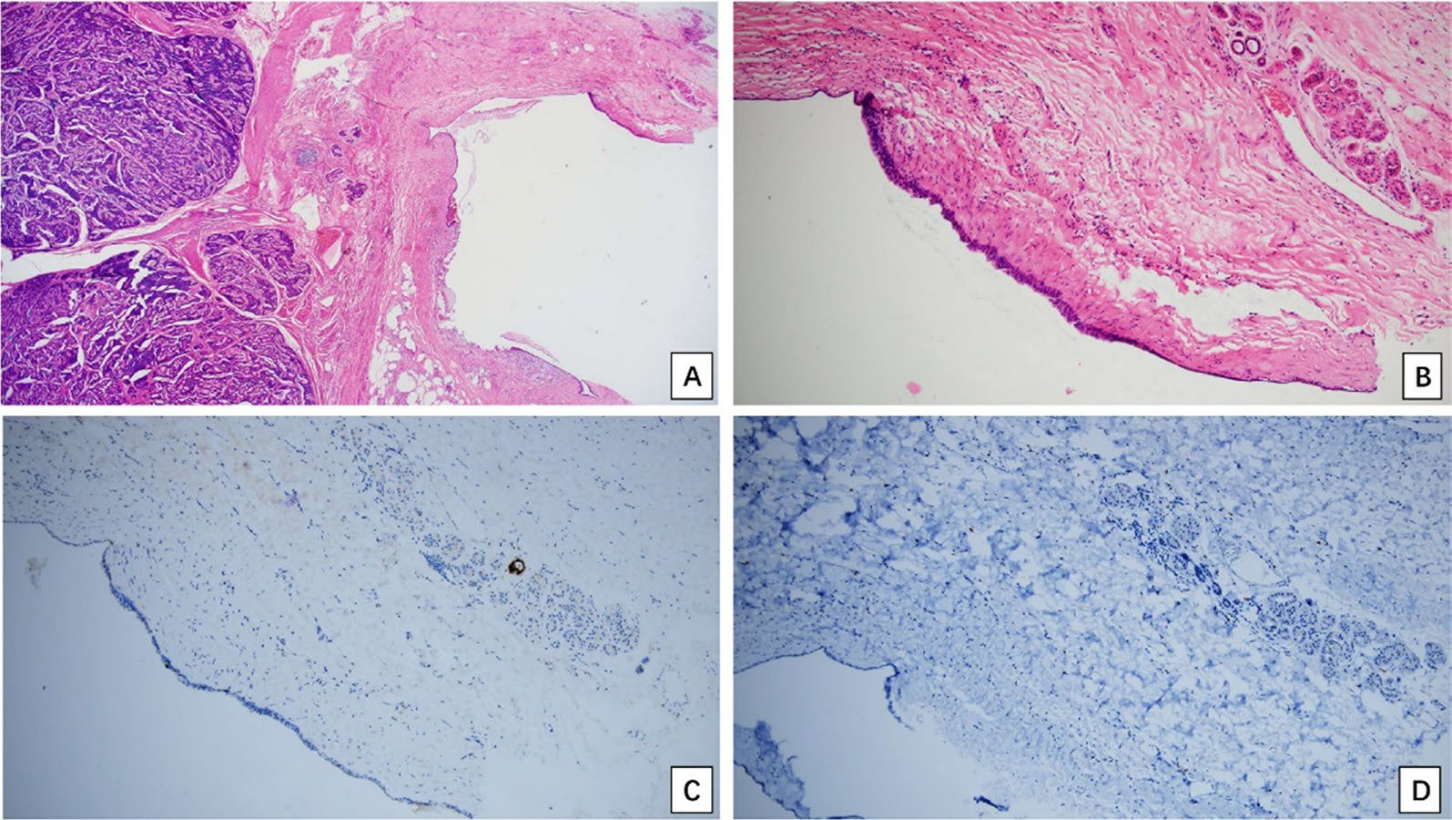
The representative morphological and immunohistochemical features of the mature cystic teratoma. **A** Adjacent to OC (left), mature cystic teratoma component was observed (right half of the **A**); **B** Magnification of the mature cystic teratoma component. The cyst wall was lined by columnar or cuboidal cells, adjacent to a few ducts and clusters of acini (top right corner of the **B**). **C** A duct is strongly positive for GCDFP15; **D** all the cells were negative for PAX8 (**A** H&E, × 40; **B** H&E, × 100; **C**, **D** IHC with hematoxylin counterstain, × 100)

Macroscopically, there was a mass measuring 2.5 cm × 1.6 cm × 1.2 cm in the left ovary, and the cut surface looked yellow and solid (Fig. 1). Histologically, the ovarian tumor exhibited two components. The main tumor (Fig. 2) was composed of cells forming parallel ribbons, cords, or trabeculae. The neoplastic cells were uniform and round to oval, with pink cytoplasm and centrally located nuclei with vesicular nucleoli. No mitosis was observed. Other focal elements adjacent to the trabecular tumor were reminiscent of a mature cystic teratoma (dermoid cyst), featuring cyst-like spaces lined by flat or cuboidal cells and a few ducts and clusters of acini, which mimicked mature skin (Fig. 3). The uterus, cervix, left fallopian tube and ovary, and all lymph nodes were negative for tumor.

Immunohistochemistry (IHC) revealed that all of the trabecular cells were strongly and diffusely positive for VIMENTIN, CD56 and synaptophysin (SYN), weakly positive for pan Cytokeratin and S-100 protein, but were negative for chromogranin (CgA), α-inhibin, TTF-1, thyroglobulin, Calretinin, WT-1 and PAX-8. The Ki-67 index was about 1%. The mature cystic teratoma component was negative for PAX-8, but the duct was positive for GCDFP15. After a comprehensive analysis of the morphological and immunohistochemical features in the tumor, imaging examination and disease history, we can rule out the metastatic low-grade neuroendocrine tumor, ovarian carcinoma, ovarian granulosa cell tumor or Sertoli-Leydig cells tumors from the ovarian carcinoid mimics. The diagnosis of primary ovarian carcinoid arising in associated mature cystic teratoma was therefore achieved according to the criteria published in the 5^th^ WHO classification of female genital tumors [7].

## Discussion and conclusions

Although ovarian carcinoid (OC) is a well-differentiated neuroendocrine tumor (NET) resembling those arising from the digestive system, there is no established nomenclature for this tumor. According to the 5^th^ WHO classification [1, 2], we can consider this case as a well-differentiated neuroendocrine tumor, Grade 1 (NET, G1) arising from an mature cystic teratoma, but the ICD-O coding is different between the two systems [1, 7]. From the limited data of primary OC, the prognosis is generally excellent, but relapse, metastasis, or even death had been reported [8–14]. Fortunately, this woman was surjected to a radical surgery and was in good condition at the current follow-up. Close follow-up, however, will continue for a long time. Therefore, we think it’s necessary to develop the current OC classification, and we recommended it would be best to follow the 5^th^ WHO classification of tumors of the digestive system [1].

Considering clinical manifestations, most patients are perimenopausal or postmenopausal females aged from 14 to 83 years (mean age: 53 years) [8]. Clinical symptoms are usually not specific, and occasionally abdominal pain, vaginal bleeding, and dysmenorrheal were reported [8]. Besides, carcinoid symptoms are seen in some insular carcinoid cases [9]. Owing to the rarity of OC, it’s important for pathologists to exclude the possibility of metastatic low-grade NET by combining imaging examinations before we achieve the diagnosis of primary OC. On the other hand, the patients, just like the present woman, undergo radical surgery and have a good outcome for ovary-confined tumor; but, given the malignant potential of the disease, they should continue to be monitored attentively.

Recently, Bidzinski et al. [10] divided OC into 4 categories based on the histopathologic characteristics: insular, strumal, trabecular, and mucinous carcinoid. (1) Insular carcinoid displays solid nests that are often punctuated by peripheral acini [11]. (2) Strumal carcinoid, intimately admixed or juxtaposed with thyroid follicles, is composed of insular or trabecular carcinoid [3]. (3) Trabecular carcinoid displays parallel ribbons, cords, or trabeculae, and the neoplastic cells are uniform and round to oval, containing pink cytoplasm and centrally located nuclei with salt-and-pepper chromatin. (4) Mucinous carcinoid is akin to an appendix carcinoid microscopically. Teratomatous elements in the ipsilateral or contralateral ovary may be present in all types of OC [12, 13]. In our study, this case was a trabecular OC.

Currently, the origin of primary ovarian carcinoid is still unclear. Vora et al. [14] suspected it arose from neural crest. Niu et al. [13] stated that the insular and mucinous types are considered as a midgut derivation, and trabecular and strumal carcinoid represent foregut or hindgut derivations.

In brief, careful morphological observation combined with appropriate ancillary histochemical kits are essential for our pathologists to approach the diagnosis of primary ovarian carcinoid arising from mature cystic teratoma, and long-term follow-up is required for this low grade ovarian neuroendocrine tumor.

## Data Availability

The datasets used analysed during the current study available from the corresponding author on reasonable request.

## Abbreviations

OC: Ovarian carcinoid
WHO: World Health Organization
NET: Neuroendocrine tumor
MRI: Magnetic resonance imaging
NSE: Neuron-specific enolase
SYN: Synaptophysin
CgA: Chromogranin
H&E: The hematoxylin and eosin
IHC: Immunohistochemistry
